# Wastewater Treatment Challenges and Circular Reuse for One Health Sustainability: A Review

**DOI:** 10.3390/ijerph23050563

**Published:** 2026-04-27

**Authors:** Imran Zafar, Shaista Shafiq, Muhammad Sohail Khan

**Affiliations:** 1 Department of Biochemistry and Biotechnology, Faculty of Science, The University of Faisalabad (TUF), Faisalabad 38000, Punjab, Pakistan; imranzafar.bio@tuf.edu.pk; 2 College of Korean Medicine, Gachon University, 1342 Seongnamdaero, Seongnam 13120, Republic of Korea; sohail@gachon.ac.kr

**Keywords:** One Health, wastewater-based epidemiology (WBE), resource recovery, multi-omics surveillance, green nanobiotechnology

## Abstract

**Highlights:**

**Public health relevance—How does this work relate to a public health issue?**

- Wastewater spreads pathogens, AMR, and emerging environmental contaminants.
- WBE and multi-omics help track public health risks in One Health systems.

**Public health significance—Why is this work of significance to public health?**

- Wastewater monitoring improves detection and management of health risks.
- Circular reuse supports water safety, nutrient recovery, and pollution control.

**Public health implications—What are the key implications or messages for practi-tioners, policy makers and/or researchers in public health?**

- Wastewater monitoring should be included in One Health policy frameworks.
- Green and circular technologies reduce long-term public health risks.

**Abstract:**

Wastewater is a complex and dynamic issue, particularly at the human–animal–environment interface, bearing biological and chemical hazards that may serve as a resource for transmission pathways for pathogens, antimicrobial resistance (AMR) determinants, heavy metals, pharmaceutical residues, per- and polyfluoroalkyl substances (PFAS), and microplastics. Rising global health issues necessitate effective wastewater treatment and advanced research to support risk-informed circular management within a one health framework, incorporating wastewater-based epidemiology (WBE), multi-omics approaches, nanobiotechnology, and green technologies. Inadequate wastewater treatment and uncontrolled discharge result in the generation of more than 380 billion cubic meters of wastewater annually worldwide, contributing to ecological degradation, the spread of AMR, and long-term toxicological risks. Despite significant advances in wastewater treatment, several challenges remain, including complex contaminant mixtures, limited detection and monitoring technologies, variable treatment efficiency, and weak regulatory and governance frameworks. This review highlights key wastewater treatment issues and presents recent advances in WBE and multi-omics approaches, such as metagenomics, resistome profiling, virome analysis, and chemical fingerprinting for contaminant monitoring and public health risk assessment. This review also examines circular reuse strategies focused on water reclamation, nutrient recovery, bioenergy production, and resource recovery, with particular emphasis on nature-based systems, hybrid biological–physicochemical treatment platforms, and green nanobiotechnology as promising approaches to improve treatment performance while minimizing environmental impacts. In conclusion, this review highlights the importance of integrated and sustainable wastewater management approaches within the One Health framework to address emerging challenges and promote environmental resilience, public health protection, and circular resource recovery.

## 1. Introduction

Wastewater is a critical environmental interface at which human, animal, and ecosystem health converge. Within the One Health framework, it functions not only as a sink for biological and chemical contaminants, but also as a dynamic pathway for the transmission of pathogens, antimicrobial resistance determinants, and emerging pollutants across environmental compartments [1]. Globally, the safe treatment of domestic wastewater remains highly uneven, with strong disparities between high-income settings and many low- and middle-income countries, where infrastructure limitations, rapid urbanization, and uncontrolled discharge continue to amplify health and ecological risks [2,3]. In this context, wastewater management must be understood not merely as an engineering or sanitation issue, but as a strategic One Health priority with direct implications for public health protection, environmental resilience, and sustainable development.

These risks are intensified by unequal wastewater treatment capacity across regions. Many low- and middle-income countries face rapid urban expansion without corresponding sanitation investment, leading to untreated discharge, greater exposure to infectious agents, and increased opportunities for antimicrobial resistance emergence and spread. Climate-related pressures, including extreme rainfall, combined sewer overflows, and drought-driven pollutant concentration, further increase the vulnerability of already stressed wastewater systems.

The worldwide volume of wastewater is enormous, reaching about 380 billion cubic meters annually, and is projected to increase further with population growth and urbanization [4]. A major public health consequence of inadequate treatment is the continued transmission of waterborne diseases, including cholera, typhoid, and hepatitis A, particularly in communities lacking safe sanitation [5,6]. Wastewater also plays an important role in antimicrobial resistance (AMR) propagation, because antibiotic residues, resistant bacteria, and resistance genes accumulate in aquatic environments and create hotspots for horizontal gene transfer [7].

The ecological consequences are equally severe. Nutrient over-enrichment, especially from nitrogen and phosphorus, drives eutrophication, harmful algal blooms, and oxygen depletion in aquatic systems [8]. At the same time, persistent and emerging contaminants such as PFAS, pharmaceuticals, toxic metals, and microplastics can remain in water bodies and sediments, where they contribute to long-term ecological disturbance and chronic toxicological risk. These combined effects make wastewater not only a source of pollution, but also a systems-level driver of interconnected human, animal, and environmental harm.

**Figure 1:**
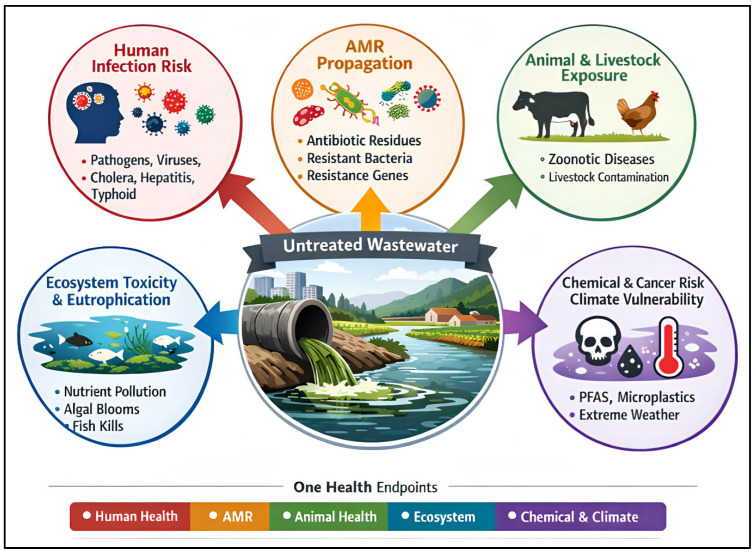
A Systems-level one health framework mapping wastewater-derived contaminants to human, animal, and ecosystem health endpoints, antimicrobial resistance propagation, and climate-linked chemical risks.

From a One Health perspective, the ultimate endpoints of wastewater mismanagement can be conceptualized across five interlinked risk domains: (i) human infection risk, (ii) AMR propagation, (iii) animal and livestock exposure, (iv) ecosystem toxicity and eutrophication, and (v) chemical and cancer risk together with climate vulnerability. These endpoints provide a functional framework for linking contaminant profiles in wastewater to concrete health and environmental outcomes, and for prioritizing surveillance and treatment strategies accordingly. The mapping of contaminants to One Health endpoints is presented in Figure 1.

The One Health framework is an influential tool for addressing wastewater issues by integrating perspectives on the benefits of improving sanitation for people, animals, and the environment. Wastewater-based epidemiology (WBE) emerged as a popular topic during the COVID-19 pandemic, with SARS-CoV-2 RNA surveillance in sewage providing early warning of outbreaks to supplement clinical data [9]. Equally, genomic wastewater surveillance across 101 countries has shown wide geographic variation in AMR genes, and sewage can serve as a global early-warning system for antimicrobial resistance, posing a threat to both human and veterinary medicine [7]. In the case of animals, enhanced wastewater treatment minimizes the impact of chronic and virulent pathogens and harmful substances in common water sources, thereby reducing the risk of spills and loss of livestock productivity [10]. Treated wastewater also benefits ecosystems by reducing contaminant loads, restoring aquatic biodiversity, and enabling the use of potable water for agriculture, thereby enhancing food security [4]. The example of cross-sectoral co-benefits depicts the need to treat wastewater not as a waste but as a strategic resource.

To transition to integrated treatment strategies, conventional technologies must be combined with innovative, sustainable, and risk-based approaches. Constructed wetlands, green buffers, and algal ponds are nature-based solutions (NBS) that have demonstrated effectiveness in removing pathogens, nutrients, and organic contaminants, while providing biodiversity and carbon-sequestration benefits. For example, hybrid constructed wetlands in Asia and Africa have demonstrated nutrient removal efficiencies of over 70% and are therefore cost-effective and robust solutions for resource-limited areas [11]. Anaerobic digestion and microbial fuel cell bioenergy recovery employing wastewater enables the conversion of liabilities into assets, producing renewable energy and minimizing greenhouse gas emissions [12]. In addition, nanobiotechnological advancements are transforming wastewater treatment paradigms. Titanium dioxide, iron oxides, silver nanoparticles, and carbon-based nanomaterials are highly reactive at the surface and antimicrobial, making them effective for removing heavy metals, pharmaceuticals, and microbial pathogens [13]. Recent advances in nanocomposite membranes and photocatalytic systems have demonstrated efficacies of up to 99% against newly emerging contaminants, at the expense of significant environmental safety and affordability concerns [14].

Despite these advances, an important conceptual gap remains: wastewater surveillance, treatment, and resource recovery are still too often treated as separate domains. This review proposes wastewateromics as an integrative One Health framework that connects contaminant source signatures, population-level surveillance, multi-barrier treatment systems, circular resource recovery, and governance pathways within a unified decision structure. In this framework, wastewater is understood not only as an environmental liability but also as a strategic infrastructure for public health protection, environmental resilience, and sustainable development.

This review synthesizes current evidence on the global wastewater burden and the major contaminant classes it contains, including pathogens, antimicrobial resistance determinants, pharmaceuticals, industrial pollutants, PFAS, microplastics, nutrients, and toxic metals. These contaminants are examined in relation to key One Health outcomes, such as infectious disease transmission, AMR propagation, ecosystem degradation, food and water insecurity, and chronic toxicological risks. The review also evaluates wastewater as a surveillance platform through wastewater-based epidemiology (WBE) and critically assesses conventional, nature-based, advanced, and circular treatment strategies, with particular emphasis on green nanobiotechnology. Finally, these dimensions are integrated into a unified wastewateromics framework that links surveillance, treatment, and governance to support sustainable wastewater management under the One Health paradigm.

## 2. Review Methodology

### 2.1. Study Design and Scope

This review was conducted as an integrative narrative review with a structured literature search to synthesize current evidence on wastewater contaminants, wastewater-based epidemiology, treatment technologies, resource recovery, and circular reuse within a One Health framework [15,16]. This approach was selected because the available literature is highly heterogeneous in terms of study design, target contaminants, treatment systems, surveillance methods, and reported outcomes, making a formal systematic review or meta-analysis less suitable for the objectives of the present paper [4,17].

### 2.2. Databases and Information Sources

The literature search was performed using Scopus, Web of Science Core Collection, PubMed/MEDLINE, and Google Scholar, together with relevant reports and guidance documents from major international organizations, to capture important policy and regulatory perspectives related to wastewater management and One Health [2,18,19,20]. Reference lists of key review papers and highly relevant studies were also screened to identify additional eligible sources [21]. For Google Scholar, screening was limited to the first 200 results sorted by relevance to improve consistency and reduce uncontrolled retrieval.

### 2.3. Search Strategy and Keywords

The search strategy was developed using Boolean combinations of controlled vocabulary and free-text terms, adapted to the syntax and indexing features of each database. Core search strings included combinations such as: “*One Health*” AND wastewater; “*wastewater-based epidemiology*” OR WBE AND surveillance; “*antimicrobial resistance*” OR AMR OR resistome AND wastewater; “*PFAS*” OR microplastics OR “*emerging contaminants*” AND treatment; “*nature-based solutions*” OR constructed wetlands OR algae OR vermifiltration; and “*nanotechnology*” OR nanomaterials OR “*green nanoparticles*” OR nanobiotechnology AND wastewater. Additional search strings combined terms such as “*resource recovery*,” “*circular economy*,” “*energy recovery*,” and “*nutrient recovery*” with wastewater-related keywords. This approach is consistent with recommended search practices for interdisciplinary environmental health reviews [16,22].

### 2.4. Time Window and Updating Strategy

The search covered the period 2015 to 2025, with greater emphasis placed on studies published from 2020 to 2025 in order to capture recent developments in wastewater-based epidemiology, antimicrobial resistance surveillance, PFAS and microplastics research, and advanced wastewater treatment technologies [9]. Earlier studies were retained when they provided foundational concepts or established evidence for conventional and emerging treatment approaches, including constructed wetlands, anaerobic digestion, microbial fuel cells, and early wastewater surveillance application [9,11,12].

### 2.5. Inclusion and Exclusion Criteria

Studies were included if they addressed wastewater contaminants, wastewater surveillance, treatment technologies, reuse, or resource recovery within a One Health, environmental health, public health, or ecosystem health context [15,17]. Eligible sources included peer-reviewed original articles, review articles, and meta-analyses, with preference given to studies demonstrating field-scale, pilot-scale, or translational relevance, particularly those using real wastewater matrices [4,23]. Studies were excluded if they focused only on synthetic model systems without practical wastewater relevance, described material synthesis without meaningful treatment application, lacked adequate methodological description, or did not contribute directly to the thematic scope of the review [16].

### 2.6. Study Screening and Selection Process

Study selection was conducted through a structured screening process based on title, abstract, and full-text assessment for relevance to the review objectives. Inclusion decisions were based on topic relevance, methodological clarity, practical applicability, and contribution to the major themes of the review. This process was used to improve transparency and reduce subjective study selection [24]. Borderline inclusion decisions were resolved through discussion among the authors to improve consistency in study selection and thematic relevance assessment.

### 2.7. Quality Assessment and Bias Control

Because the included literature covered multiple disciplines and study designs, a formal risk-of-bias scoring tool was not applied. Instead, study quality was appraised qualitatively using criteria including relevance to the review objectives, clarity of methods, use of real wastewater or field-applicable conditions, reporting of measurable outcomes, translational value, and discussion of implementation limitations [25,26]. Greater interpretive weight was given to field-scale and pilot-scale studies, followed by systematic reviews, meta-analyses, and well-designed laboratory investigations using real wastewater. For nanotechnology-related studies, additional attention was given to material stability, reuse potential, leaching risk, energy demand, and environmental safety, in line with safe-and-sustainable-by-design and life-cycle thinking [19,27].

### 2.8. Data Extraction and Synthesis

Data were extracted on wastewater type, contaminant category, surveillance or treatment approach, scale of application, key findings, economic or implementation considerations, and relevance to One Health. The evidence was synthesized thematically rather than statistically and organized around major domains, including contaminant sources and signatures, wastewater-based epidemiology, treatment technologies, nature-based systems, nanobiotechnology, and circular resource recovery [28,29]. A quantitative meta-analysis was not attempted because of substantial heterogeneity in study designs, target pollutants, performance indicators, and reporting formats across the included literature [30].

### 2.9. Limitations of the Methodology

This integrative narrative review lacks the standardization of a systematic review and is subject to literature heterogeneity, inconsistent cost reporting, and limited full-scale validation, particularly for emerging technologies [31,32]. Many approaches rely on laboratory or pilot-scale evidence, restricting large-scale applicability. These limitations were addressed by prioritizing practically relevant studies and critically assessing implementation constraints [33].

## 3. The One Health Burden of Wastewater: Contaminants and Sources

**Figure 2:**
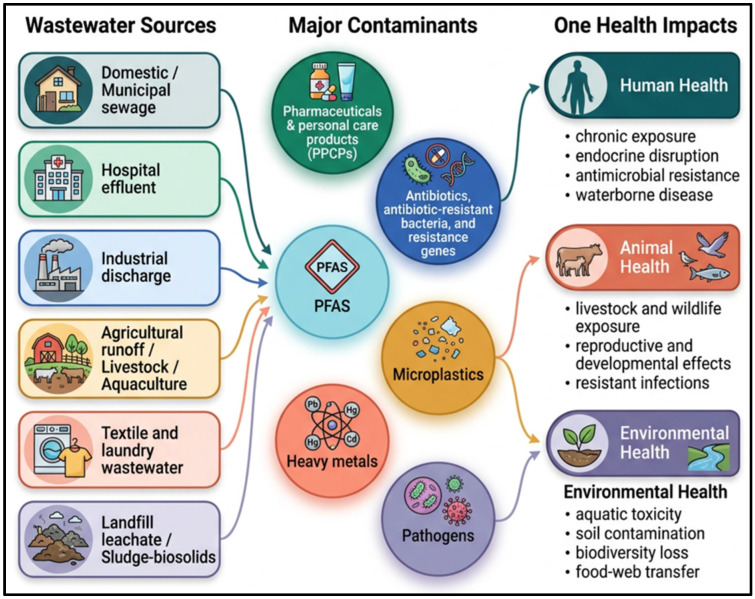
The One Health Burden of Wastewater: Contaminants, Sources, and Exposure Pathways.

Wastewater is a heterogeneous mixture of chemical, biological, and physical contaminants, with compositions that vary with source, land use, and technology, producing a unique signature of the source that can be diagnostically important through the lens of One Health. The pollutants that contribute to wastewater contamination originate from diverse municipal and industrial sources and discharge chemical, biological, and emerging pollutants that permeate aquatic and terrestrial environments. The pathways of dissemination and ecological impacts of these contaminants are illustrated in Figure 2, which highlights the interconnected pathways by which wastewater pollutants enter the environment. Municipal streams mirror human metabolism and behavior, including urea, creatinine, bile salts, steroid hormones, lifestyle tracers (e.g., caffeine, nicotine), and diverse pharmaceuticals, and they also carry pathogens (e.g., *E. coli*, noroviruses) and antibiotic resistance genes (ARGs). Based on these signatures, WBE can be used to monitor both infectious diseases and population exposures, and recent progress has shown that it can do so using rapid, near-source tests and strong correlations with outbreak dynamics [34,35].

Industrial effluents have sector-related fingerprints: textile dyeing releases chromophoric azo dyes that contain metals (Cr, Cu) and increase the BOD/COD; pharmaceutical/chemical production releases active ingredients, solvents, and waste; mining and plating contain Hg, Cd, As, and Cr (VI) [36,37]. The newly formed PFASs persist despite traditional treatment and are associated with immunotoxicity, lipid dysregulation, and increased cancer risk [38,39]. Nutrient surpluses (N, P) from agricultural and livestock runoff contribute to eutrophication and coastal hypoxia, and manure increases the selection and mobility of ARGs by introducing zoonotic pathogens and veterinary drugs [40].

Wastewater from cities and industries contains a broad range of pollutants, including organic substances, heavy metals, nutrients, and emerging contaminants, all of which pose significant ecological and health threats. Table 1 summarizes the distribution, representative compounds, and effects of these contaminant classes, providing insight into their occurrence and significance in wastewater treatment plans. Disinfectants, cytotoxics, and multidrug-resistant organisms (MDROs) are the primary targets of healthcare wastewater, increasing the spread of ARGs and imposing stress on municipal WWTPs not designed to handle such loads [41,42]. Stormwater and urban runoff intermittently carry metals (Pb, Zn, Cu), PAHs, nutrients, pathogens, and large loads of microplastics, making them important pathways of contaminant transfer to surface waters [43]. Taken together, these source-resolved fingerprints are useful for proactive monitoring and control. High-throughput sequencing and metagenomics can identify ARG reservoirs in WWTPs that treat approximately half of the world’s sewage [44]. In parallel, mass spectrometry and biosensors can quantify micropollutant burdens to support targeted treatment and source management strategies.

## 4. Monitoring, Analytics, and Wastewater-Based Epidemiology

Wastewater-based epidemiology (WBE) is an epidemiology that has changed the traditional surveillance of population health by offering real-time data on the movement of pathogens, antimicrobial resistance, environmental exposures, and community-level behavior, which is a fast, objective, unbiased addition to traditional case-based systems of health surveillance [61]. The sensitivity of WBE was high during the COVID-19 pandemic, where viral RNA indicators in wastewater tended to detect the reported cases of the disease days to weeks before, allowing detection of an outbreak and response earlier [9,62]. In addition to infectious diseases, it is extensively applied to track pharmaceuticals, pesticides, illicit drugs, and industrial pollutants, which supports its applicability in the context of One Health and the more general public health systems [34,35]. This broad applicability can be seen as a strength of WBE: the capability of population-level inferences based on a single aggregated environmental sample, especially in those environments where clinical surveillance is delayed, incomplete, or resource-consuming. Most recently, the international community has been working on formalizing WBE into public health systems, with the World Health Organization (WHO) in 2024 providing guidance on how to incorporate wastewater and environmental surveillance into standard decision-making based on routine sampling, data interpretation, and ethical governance [63]. This institutional change underscores how WBE has evolved from an experimental tool to a structured surveillance tool, where detection potential is not the sole key element but also the significance of standard interpretation and practical utilization of information in a variety of different public health settings.

These principles make WBE not only a research instrument and an early-warning system, but also a supplementary surveillance pillar alongside clinical and syndromic data, especially in low-resource and fast-developing settings. WBE has analytical sophistication, based on its tiered methodologies that combine chemical and biological methods. Targeted analysis using established platforms such as liquid chromatography–tandem mass spectrometry (LC–MS/MS) enables quantitative measurement of pharmaceuticals, disinfectants, and other priority contaminants in wastewater [64]. Omics-based methods have transformed biological surveillance; metagenomic resistome profiling can monitor the abundance of ARGs in wastewater, virome research can identify the circulation of respiratory and enteric viruses, and mobilome mapping can identify pathways by which horizontal gene transfer promotes AMR spread [65]. Combined, these measures create a multidimensional surveillance platform to detect known and emerging threats, highlighting the integrative nature of WBE. From a discussion perspective, the integration of targeted chemical analytics with omics-based biological surveillance substantially strengthens the interpretive value of WBE, because it allows simultaneous assessment of exposure, transmission dynamics, and resistance dissemination. This multidimensionality is especially relevant to One Health frameworks, where human health, environmental contamination, and microbial evolution are closely interconnected rather than operating as isolated domains.

WBE is based on sound sampling design, data flow, and governance to guarantee a credible interpretation of public health. Passive samplers can be used in low-cost, long-term monitoring of contaminants, whereas flow-proportional or time-composite sampling is necessary to monitor diurnal variability and detect outbreaks [66]. WBE has proven useful in large-scale applications, such as national and city-wide surveillance initiatives, to monitor trends in pathogen circulation and antimicrobial resistance in high- and low-resource environments [67,68]. The process of turning wastewater data into intelligence that can be acted on needs powerful QA/QC processes, normalization with fecal indicators including pepper mild mottle virus (PMMoV), and sophisticated statistical models to consider sewer dynamics and dilution effects of rainfall [69,70]. These aspects indicate that WBE reliability is not just determined by the sensitivity of the analytical technique, but also sampling representativeness, normalization procedures, and system context. Thus, it can be seen that WBE can be understood as a context-specific surveillance instrument in which standardized methodology and proper fit with epidemiological goals are critical to derive similar and useful public health information.

**Figure 3:**
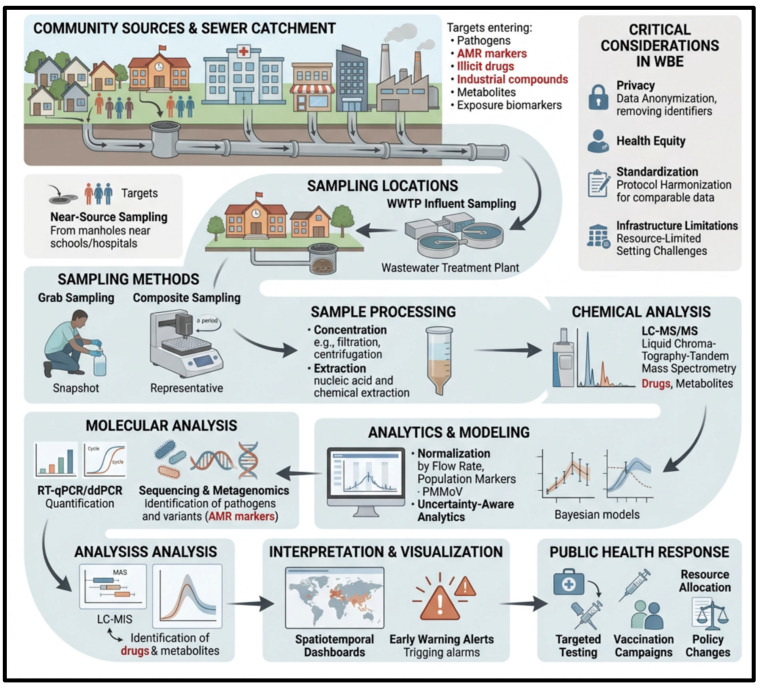
Workflow of Wastewater-Based Epidemiology for Community Health Monitoring and Public Health Surveillance.

WBE needs explicit connections between detection and intervention to operate as a decision-support system, based upon specified thresholds, predictive lead times, and governance frameworks that dictate who needs to act. Its applicability relies on its ability to differentiate between actual signals and false positives due to temporary population changes, rainfall-related overflows, or industrial discharges, which may need to be verified by environmental and clinical data. WBE programs require a coordinated effort of utilities, public health agencies, and policymakers with set protocols of escalation, communication, and response in place to ensure the system remains a part of a multidisciplinary approach, not undertaken alone. With the growth of WBE, ethical issues start playing a vital role in this area, such as proper use of data, protection of the privacy of the community, prevention of stigmatization, and open governance in accordance with international standards, as observed in Figure 3. Finally, its success lies in the ability to balance the analytical accuracy, relevance to the health of the population, and responsible governance through an integrated surveillance system.

## 5. Advances in Wastewater Treatment Technologies: Nature-Based, Biological, and Advanced Approaches

The treatment of wastewater is moving towards a more circular economy model of resource recovery and sustainability, rather than merely removing pollution, which is in line with the principles of One Health. Primary and secondary treatment is effective at removing bulk organic matter and solids, but is not sufficient to remove emerging contaminants like pharmaceuticals, pesticides, endocrine-disrupting compounds, PFAS, and AMR determinants [71]. To overcome these deficiencies, tertiary and quaternary treatments are used as polishing processes to enhance the quality of effluent using a treatment train system that incorporates primary/secondary removal, tertiary nutrient and pathogen removal, and advanced quaternary removal of micropollutants, then followed by reuse or discharge. Traditional tertiary treatment approaches, including filtration, activated carbon adsorption, chlorination, ozonation, and UV disinfection, provide greater safety, but adsorption with activated carbon encounters several challenges, including breakthrough and regeneration [72]. Advanced oxidation processes (AOPs) also degrade recalcitrant pollutants, but are limited by energy requirements and by-products. Nature-based systems (constructed wetlands, algal and duckweed ponds, vermifiltration) provide low-energy, sustainable wastewater treatment with resource recovery, while emerging technologies (microbial fuel cells, mycoremediation, bioelectrochemical systems) enable simultaneous pollutant removal and energy/bioproduct generation, as shown in Table 2.

Wastewater treatment and advanced treatment systems are increasingly shaped by stronger regulatory frameworks that promote micropollutant removal, resource recovery, and environmental protection. Membrane technologies, including microfiltration, ultrafiltration, nanofiltration, and reverse osmosis, are widely used for micropollutant and PFAS removal, with reverse osmosis showing high removal efficiency but remaining limited by high energy demand, membrane fouling, and brine disposal challenges [73]. The EU Urban Wastewater Treatment Directive (2024/3019) introduces extended producer responsibility (EPR), requiring pharmaceutical and cosmetic industries to contribute to treatment costs and formally integrating quaternary treatment into compliance systems [74,75]. PFAS treatment remains challenging because available technologies involve important trade-offs: nanofiltration and reverse osmosis provide broad removal capacity, whereas activated carbon and ion exchange are more selective, and all approaches may generate secondary waste requiring further management. Overall, policy and technology developments are driving a shift toward integrated, multi-barrier systems that combine membranes, advanced oxidation processes, adsorption, and nature-based solutions, positioning quaternary treatment as a key regulatory standard for sustainable water reuse and environmental protection [75].

**Table 2:** Nature-based and biological wastewater treatment approaches, showing treatment mechanisms, target pollutants, typical performance, scale of application, and sustainability benefits.

Method	Green Principle	Wastewater Type/Scale	Key Findings (Typical)	References
Constructed wetlands (horizontal/vertical/hybrid)	Plant–microbe systems; passive treatment, low chemicals	Municipal/domestic; pilot → full scale	High BOD/TSS/N/P removal; robust seasonal performance; scalable designs exist	[76]
Floating treatment wetlands (FTW)	Emergent plants on floating mats; roots support biofilm	Stormwater/retention ponds; field	Significant TN/TP reductions; species & harvest timing affect removal	[77]
Duckweed/Lemnaceae systems	Rapid plant uptake; biomass valorization	Municipal/agricultural; pilot	Very high N & P uptake; harvestable protein/starch biomass	[78]
High-rate algal ponds (HRAP)/algal raceways	Algal-bacterial photosymbiosis; solar O_2_	Domestic/municipal; pilot → hectare scale	Good nutrient removal + biomass for energy/fertilizer; low energy input in sunny climates	[79]
Vermifiltration/biofilters (earthworm-assisted)	Earthworm–microbe synergism; physical filtration	Domestic/agricultural; pilot → full scale	COD/BOD and pathogen reductions; low sludge production; low energy	[80]
Bioflocculants (microbial biopolymers)	Microbial polymers replace chemical flocculants	Industrial/municipal; lab → pilot	Efficient solids & metal removal; biodegradable alternatives to alum/Fe salts	[81]
Immobilized enzymes (e.g., laccase)	Biocatalysis for dyes/phenols; immobilization for reuse	Textile/pharma; lab → continuous packed bed pilot	High decolorization; long operational stability in immobilized form	[82]
Integrated multitrophic aquaculture (IMTA)	Multi-trophic uptake (algae, plants, animals)	Aquaculture; pilot	Nutrient removal + co-product biomass (fish, seaweed)	[83]
Bioelectrochemical systems/Microbial Fuel Cells (MFCs)	Microbial oxidation produces electrons (electricity) + treatment	Domestic/organic waste; lab → pilot	Simultaneous COD removal & electricity generation (low power); scaling challenges	[84]
Mycoremediation (fungal bioreactors/fungal biosorption)	Fungi (white-rot, marine) produce ligninolytic enzymes; biosorption	Dye/industrial/tough organics; lab → pilot	Effective degradation of dyes, phenolics, and some POPs; good biosorption of metals	[85]
Bivalve (oyster/mussel) bioremediation/bioextraction	Filter feeding + biodeposition increases denitrification & harvest removal	Coastal/estuarine; restoration & aquaculture scale	Oysters can remove/transform nitrogen via assimilation, denitrification enhancement, and harvest	[86]
Intermittent sand filters (ISF)/soil aquifer treatment (SAT)	Physical filtration + biofilm nitrification/denitrification in soil	Decentralized municipal community	High BOD/TSS removal; robust pathogen reduction; mature technology	[87]
Rotating biological contactors (RBC) & biofilm reactors	Attached-growth biofilms on media; low energy	Municipal/industrial; full scale	Reliable BOD/N removal; compact footprint; used worldwide	[88]
Phycoremediation—photobioreactors & Rotating Algal Biofilm (RAB)	Algal biofilm cultivation (attached) for nutrient uptake	Industrial/municipal; pilot	Easier harvesting vs. suspended algae; good nutrient capture	[89]

### 5.1. Comparison of Conventional and Advanced Wastewater Treatment Technologies

The comparative analysis of the wastewater treatment technologies indicates blatant performance and application scales of conventional, advanced, nature-based, and emerging systems. As described in Table 3, the most developed and affordable method of bulk removal of BOD, COD, and suspended solids remains conventional biological processes, which are necessarily limited in the removal of hard-to-treat micropollutants like PFAS, pharmaceuticals, and determinants of antimicrobial resistance. Conversely, tertiary and membrane-based processes and advanced oxidation technologies are more appropriate in high-quality polishing and water reuse applications, but are limited by increased energy consumption and cost, fouling, and concentrate management challenges. Nature-based systems are low-energy and ecologically co-beneficial but highly site-sensitive based on land requirements, climate sensitivity, and variable trace contaminant removal. Nano-enabled and hybrid systems are growing in theory with high removal potential, but remain limited by scale-up uncertainty, lifecycle risks, and economic feasibility. Table 4, complementarily, gives representative examples of technologies and asserts that integrated hybrid and multi-barrier systems that combine biological, membrane, and resource-recovery methods are the most robust and adaptable performers, though with a higher level of complexity of operations and system integration demands.

### 5.2. Bioenergy and Resource Recovery Approaches in Wastewater Treatment

The idea of wastewater management is moving beyond relying on the removal of pollutants to more comprehensive principles of the circular economy, such as the generation of energy, recovery of nutrients, and valorization of resources. Anaerobic membrane bioreactors (AnMBRs), expanded granular sludge bed reactors, and algal-bacterial consortia have been demonstrated to be very effective in the removal of organic matter, generation of biogas that contains methane, and also in the reduction of sludge generation. Simultaneously, various methods based on nutrient recovery, including struvite crystallization and partial nitritation-anammox (PN/A), allow extracting nitrogen and phosphorus as beneficial fertilizers. Through the integration of such technologies, the wastewater treatment plants may become energy-neutral or even energy-positive, thus directly supporting the targets of renewable energy sources and sustainable agriculture.

**Figure 4:**
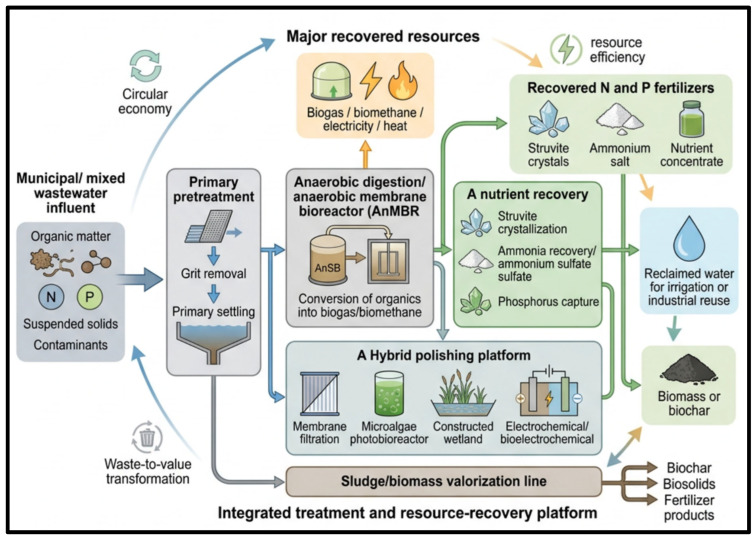
Integrated bioenergy production, nutrient recovery, and hybrid treatment platforms for energy-neutral circular wastewater management.

As Figure 4 (conceptual) demonstrates, the contemporary wastewater treatment systems are being redesigned as integrated resource recovery systems that integrate wastewater inflow, biological conversion, nutrient capture, and sophisticated polishing processes into a single circular management system. Wastewater with organic matter, nitrogen, phosphorus, and other contaminants can be treated through interlocking treatment modules whereby the organic fraction is transformed to biogas or biomethane via anaerobic processes, whereas the nutrient-rich streams can be treated to recover nitrogen and phosphorus in forms that can be used as fertilizers, i.e., in forms of struvite and ammonium. Simultaneously, hybrid treatment systems, such as membrane, algal-bacterial, electrochemical treatment, and nature-based polishing systems, also enhance the quality of effluent and can be used to reuse the water. The figure also highlights the circular relationship between treatment and valorization by illustrating that energy, nutrients, reclaimed water, and products derived from the biomass could be recovered from the wastewater streams, thus decreasing the generation of waste and enhancing the sustainability of the entire process.

Along with individual systems, a combination of bioenergy systems and ecological and nanotechnology-enhanced systems is producing hybrid treatment platforms. These can be AnMBRs with constructed wetlands, microbial electrochemical cells with anaerobic digestion, and biochar-based adsorbents with biological polishing processes. Such hybrid structures allow synergistic interactions between biological conversion, electrochemical conversion, and nature-based or nanomaterial-based polishing processes. The benefits of these hybrid systems are synergistic degradation of micropollutants, decreased greenhouse gases, and decentralized applicability in urban and peri-urban environments. Moreover, the integrated designs facilitate the shift in a linear model of treatment to a circular wastewater biorefinery model where several value-added products are produced at the same time with reduced energy usage and environmental impact. Table 5 shows the comparative analysis of these innovative approaches with emphasis on their performance efficiencies, recovery potential, and sustainability trade-offs, providing a complete guide to the next-generation approaches to choosing solutions in the field of circular wastewater management.

## 6. Nature-Based Systems (NbS) with Modern Upgrades

With current technological advances, Nature-Based Systems (NbS) have revolutionized sustainable water management by integrating natural ecological processes with modern technological interventions. Conventional NbS, such as constructed wetlands and riparian buffers, were modified into multifunctional, designed solutions that address the entire gamut of water-related problems. For example, Constructed Wetlands 2.0 now employs intensified media (e.g., ceramsite) and sophisticated aeration methods that increase microbial activity, oxygen transfer, and the degradation of organic matter and micropollutants [98]. Step-feed designs have also enhanced nitrogen removal efficiency by maximizing microbial pathways and by using hybrid wetlands, which have been observed to perform better in treating industrial and agricultural wastewater through a combination of vertical and horizontal flows. Equally, Riparian Buffers have been modified into biochar-amended soils, which adsorb nutrients more effectively, improve long-term soil fertility, and reduce diffuse agricultural pollution. These systems not only prevent runoff from entering water bodies but also support biodiversity, which is critical to preserving ecological integrity. In coexistence with these ecological designs, hybrid NbS offers customized solutions for various wastewater streams, underscoring that landscape-based interventions are also part of broader water security plans.

**Figure 5:**
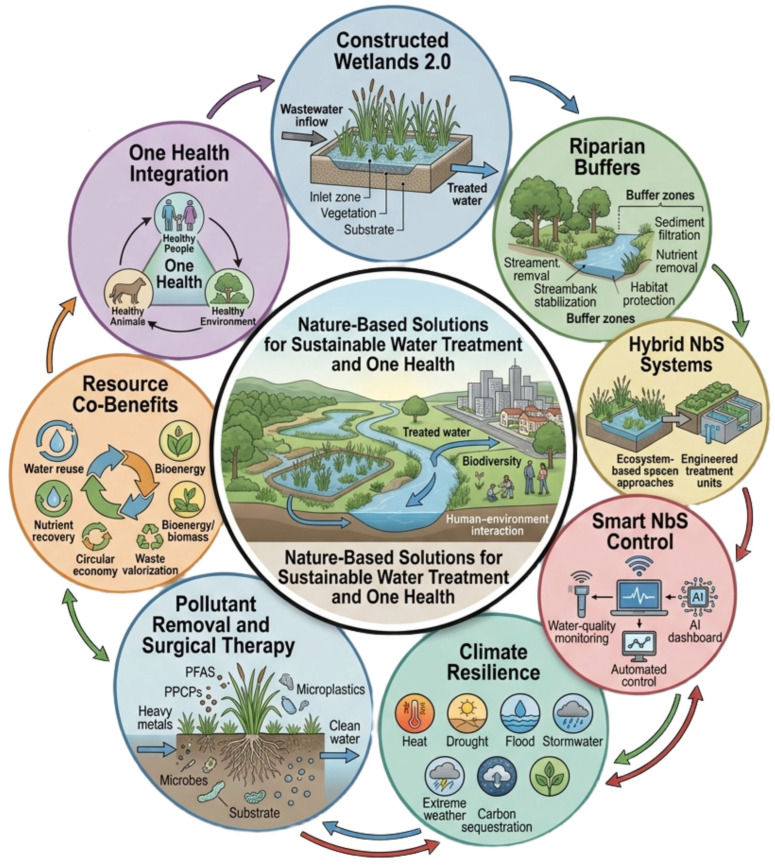
Advanced Nature-Based Systems (NbS) integrating constructed wetlands, riparian buffers, hybrid ecological designs, smart IoT/AI controls, and climate-resilient strategies to enhance pollutant removal, resource recovery, biodiversity conservation, and One Health sustainability.

In addition to these structural and ecological gains, the integration of intelligent control systems and climate-adaptation functions has elevated NbS to a higher level of performance. With the help of IoT sensors and AI-based optimization, Smart NbS Control enables real-time monitoring of water quality, adaptive load control, and event-driven aeration, which, in turn, improve treatment process efficiency and help reduce energy use. This layer of digitalization ensures that NbS are not only affordable and eco-friendly but also technically competitive with traditional systems. Meanwhile, NbS are also being identified as climate-resilience instruments, alleviating urban heat island effects, reducing the risk of flooding during extreme precipitation, and minimizing CSOs. Their multifunctionality extends to Resource Co-Benefits, including carbon sequestration, soil enrichment, and habitat creation, which directly tie to One Health Integration and reinforce biodiversity and human-environmental well-being. All of these innovations underscore how NbS have evolved, now relying on technology as a dynamic framework rather than on passive, nature-inspired ones that provide water-quality benefits, ecosystem services, and societal resilience in a single integrated package, as in Figure 5.

## 7. Nanobiotechnological Breakthroughs and Green-Synthesized Nanoparticles for Wastewater Treatment

**Figure 6:**
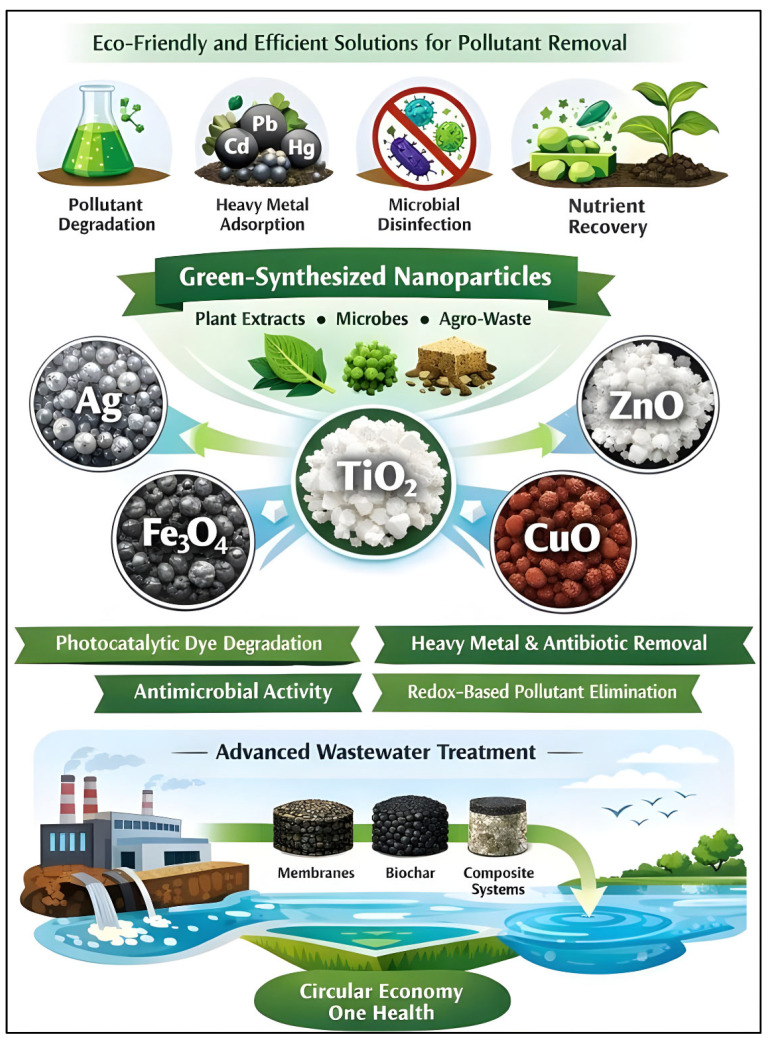
Integrated nanobiotechnological pathways enabled by green-synthesized nanoparticles for multifunctional wastewater treatment within a circular economy and One Health framework.

Nanobiotechnology has emerged as a promising but still unevenly mature option in wastewater treatment. Green-synthesized nanoparticles and nano-enabled composites can enhance pollutant degradation, adsorption, disinfection, and nutrient recovery, particularly in industrial polishing steps and other high-value applications, as detailed in Figure 6. However, the evidence base remains dominated by laboratory and early pilot studies, and large-scale implementation is constrained by material cost, recovery and reuse challenges, nanotoxicity concerns, and uncertainties in lifecycle performance. In this review, these technologies are therefore discussed as innovative adjuncts to—not replacements for—established treatment trains within a circular One Health framework. Table 6 provides a consolidated overview of representative developments and their current application scope.

The key benefit of such green synthesis pathways is that they align with the principles of green chemistry, many of which rely on low-energy reactions, benign solvents, and renewable feedstocks, thereby reducing the overall environmental impact of creating nanomaterials. Nevertheless, to realize their potential, rigorous lifecycle assessments (LCAs) are necessary to determine their sustainability from synthesis through disposal, and to ensure they provide a net environmental benefit over both traditional treatments and chemically synthesized nanomaterials. They should also be contextualized in terms of their application, as they have the potential to be high-value for treating or polishing industrial effluent, but their scalability and economic feasibility for large volumes of municipal wastewater in resource-constrained locations require further research and innovation.

Green-synthesized nanoparticles are especially appealing because natural reducing and stabilizing agents are incorporated into them, thereby improving stability, reducing aggregation, and lowering the risk of secondary contamination. Sustainable production of nanoparticles with unparalleled catalytic and antimicrobial activities is possible by the use of plant-derived extracts that are rich in polyphenols, proteins, polysaccharides, and microbial metabolites. An example of such nanoparticles is silver nanoparticles from Acacia cortex, which have been shown to be effective for dye degradation and bacterial inactivation, whereas ZnO nanoparticles prepared from algal biomass exhibit visible-light-driven photocatalysis of textile wastewater. On the same note, biogenic TiO_2_ and CuO nanoparticles have shown promise in removing toxic metals and antibiotics from industrial effluents. Their introduction into composite systems, membranes, and biochar support systems has contributed to their recovery, reuse, and applicability in large-scale operations. The integration of nanobiotechnology with green synthesis pathways positions it as a pillar of next-generation wastewater management systems, despite current challenges such as nanoparticle stability, scalability, and environmental persistence. Therefore, the performance values reported for green-synthesized nanoparticles and nanocomposites should be interpreted primarily as evidence of technical potential rather than proof of immediate large-scale applicability, particularly where results are derived from synthetic matrices, short-duration experiments, or early pilot conditions as detailed in Table 6.

### 7.1. Green Nanocomposites and Photocatalytic Membrane Technologies

Green nanocomposites have attracted significant attention as multifunctional materials for wastewater treatment because they integrate various active components, including metal oxides, carbonaceous materials, biochar, nanocellulose, and carbon quantum dots. The hybrid systems consist of the individual catalytic, adsorptive, and antimicrobial characteristics of nanomaterials with the stability, renewability, and environmental friendliness of natural supports. For example, magnetic biochar composite offers a high surface area for pollutant adsorption and can be easily separated and reused, thereby enabling effective elimination of dyes, heavy metals, and pharmaceutical residues. Similarly, nanocellulose membranes, a renewable biomass-derived material, have remarkable mechanical strength and tunable surface properties, making them suitable for the selective removal of contaminants in both domestic and industrial effluents. Carbon quantum dots (CQDs) derived from biomass have emerged as visible-light-responsive photocatalysts for degrading persistent organic micropollutants and as nanosensors for environmental monitoring of water quality. Recent reports have described the creation of green nanocomposites, such as rGO/TiO_2_, CuO@A-TiO_2_, and bio-waste-based FeO/ZnO, which exhibit excellent photocatalytic activity and enhanced heavy metal removal compared to their individual counterparts, as detailed in Table 7. Photocatalytic membrane reactors (PMRs) combine membrane filtration with photocatalysis, using green-synthesized nanoparticles to enhance pollutant degradation while reducing fouling and extending membrane life. Integrated with advanced oxidation processes (e.g., UV/H_2_O_2_, photo-Fenton, persulfate), they effectively remove pharmaceuticals, dyes, and resistant microbes in tertiary treatment with lower chemical and energy input. Sustainability is improved using biopolymer- and waste-based supports such as chitosan, clays, and agricultural residues, while advanced nanocomposites (e.g., seaweed–zeolite, Pd/Fe_3_O_4_, TiO_2_–silica) enable efficient removal of metals, antibiotics, and dyes, positioning PMRs as key next-generation wastewater treatment systems.

### 7.2. Nano-Bio Hybrids and Renewable-Energy-Driven Advanced Processes

Nano-bio hybrids are the vanguard of sustainable nanobiotechnology, as nanomaterials are combined with biological catalysts, enzymes, and biotemplated materials for selective adsorption, catalytic transformation, and enhanced biodegradation. Immobilization of enzymes such as laccase and peroxidase onto nanoparticle supports enhances stability, reuse, and catalytic selectivity for the degradation of dyes, phenols, pharmaceuticals, and other recalcitrant organic contaminants. Likewise, metal–organic frameworks biomass-templated (bio-MOFs) are characterized by ultrahigh surface area, tunable porosity, and high abundances of functional groups for the selective capture of pharmaceuticals, heavy metals, and dyes (in laboratory and pilot-scale environments). The derived agricultural residues into graphene oxide (GO) and reduced graphene oxide (rGO) not only enhance adsorption and electronic transfer characteristics but also improve compatibility with solar-driven photocatalytic reactions. These systems not only remove pollutants but also enable resource recovery, making them viable for wastewater management in a circular economy.

Simultaneously, renewable-energy-driven advanced processes are becoming viable as a means of meeting the energy requirements of traditional treatments. The contact area, light harvesting, and throughput of aerogels and three-dimensional porous nanostructures are also higher than those of slurry photocatalysts, and they are easily separable and reusable. By incorporating green-synthesized nanoparticles into membranes and PMRs, it is possible to perform filtration, pollutant degradation, and fouling control, thereby extending membrane lifespans. Processes that can be powered by renewable energy sources, including photovoltaic-assisted electrocoagulation (PV-EC), can be used to treat wastewater independently of the grid, with a lower carbon footprint, whereas solar-based AOPs, including photo-Fenton, UV-H_2_O_2_, and persulfate activation, exhibit high levels of dye, pharmaceutical, and pathogenic microorganism removal. Else (e.g., Fe-doped TiO_2_ and Fe_3_O_4_–TiO_2_) magnetic photocatalysts are also well-suited for easy magnetic recovery, thereby increasing efficiency and reusability. These nanobiohybrid systems and renewable-energy-driven processes together offer scalable, low-impact, and versatile opportunities to remediate wastewater while following the principles of green chemistry and One Health. Table 8 provides a concise history of recent developments, synergies, wastewater uses, and pollutant removal performance, reflecting their increasing importance in next-generation water treatment technologies.

## 8. Green Nanomaterials for Water and Wastewater Treatment: One Health Perspectives, Cancer Prevention Potential, and Toxicological Insights into Lead (Pb) and Chromium (Cr) Carcinogenicity

Green nanomaterials have become widely acknowledged as environmentally friendly agents of water and wastewater purification because they are produced in an eco-friendly manner, possess multifunctional reactivity, and are not extremely toxic, as shown in Table 9A. Plant, microbial and waste-based, as well as other sources, including chitosan, silver, gold, ZnO, TiO_2_, copper nanoparticles and carbon based nanostructures, have high adsorption, photocatalytic, antimicrobial and degradation properties in the removal of heavy metals, dyes, pesticides and pathogens, thereby mitigating exposure to car Simultaneously, Table 9B underlines the toxicological load of Pb (lead) and Cr (chromium), especially Cr(VI), capable of causing carcinogenesis due to oxidative stress, DNA damage, epigenetic dysregulation, and activation of the main signaling pathways (e.g., p53, NF-kB, JAK/STAT3), with high relations to lung cancer, breast cancer Collectively, these results highlight a two-fold system whereby green nanomaterials can reduce carcinogenic contaminants in water systems as well as the underlying toxicological pathways of the persistent metals, merging environmental remediation, cancer risk mitigation, and One Health principles into a single model.

## 9. Future Directions

Future studies should move beyond fragmented wastewater treatment approaches toward integrated wastewateromics systems that combine contaminant tracking, wastewater-based epidemiology, multi-omics surveillance, treatment optimization, and circular resource recovery within a unified One Health framework. Standardized protocols for sampling, normalization, quality assurance, and data interpretation are needed to improve comparability across settings and strengthen the public health value of wastewater-derived signals. At the same time, WBE systems should be more clearly linked to intervention thresholds, predictive lead times, and governance pathways so that surveillance outputs can support timely and coordinated responses by utilities, health agencies, and policymakers.

At the treatment level, future progress should prioritize full-scale validation of hybrid and multi-barrier systems under real wastewater conditions, particularly those integrating biological treatment, membrane separation, advanced oxidation, and nature-based polishing. Greater attention is also needed for resource recovery, climate resilience, and the responsible development of green nanobiotechnology, including lifecycle assessment, nanotoxicity, leaching risk, recovery, and long-term performance evaluation. Ultimately, stronger policy integration, equitable infrastructure investment, and collaboration among engineers, microbiologists, toxicologists, epidemiologists, and environmental planners will be essential to develop wastewater systems that are technically effective, economically feasible, environmentally sustainable, and socially acceptable.

## 10. Conclusions

This review demonstrates that wastewater is not merely a disposal problem, but a critical operational interface within the One Health framework, linking human, animal, and environmental health through interconnected pathways of exposure, surveillance, and intervention. Wastewater contains a complex mixture of pathogens, antimicrobial resistance determinants, nutrients, pharmaceuticals, PFAS, microplastics, and toxic metals that contribute to infectious disease transmission, AMR dissemination, ecosystem degradation, and long-term toxicological risk, particularly where treatment infrastructure is inadequate. At the same time, wastewater also represents a valuable source of actionable information and recoverable resources when approached through integrated monitoring and circular treatment systems.

This review also highlights a clear transition from conventional linear treatment models toward integrated, multi-barrier, and circular wastewater management systems. Advances in wastewater-based epidemiology, metagenomics, resistome profiling, virome analysis, nature-based systems, bioenergy recovery, membrane technologies, advanced oxidation, and green nanobiotechnology are expanding opportunities for safer treatment, resource recovery, and reclaimed-water reuse. However, many emerging approaches remain limited by heterogeneous evidence, incomplete full-scale validation, variable cost reporting, implementation complexity, and safety concerns. In this context, wastewateromics offers a unifying framework that connects contaminant signatures, real-time surveillance, treatment design, circular recovery, and governance within a single decision-support model. With continued technological innovation, standardization, responsible governance, and equitable implementation, wastewater systems can evolve from passive sanitation infrastructure into active tools for health security, environmental resilience, and sustainable circular development.

## Figures and Tables

**Table 1:** Major contaminant classes in municipal and industrial wastewater, including representative compounds, typical occurrence ranges, and principal environmental and public health significance.

Contaminant Class	Representative Materials	Typical Occurrence in Wastewater	Why It Matters	Citations
Heavy metals/metalloids	Al, Ni, Pb, Cr, Hg, Cu, Zn, As, Cd, Li	Municipal WWTP effluents (Türkiye, 15 plants): Hg ND–0.42 µg/L (mean 0.12), Pb 0.02–28.93 µg/L, Ni 0.07–123.41 µg/L (mean 15.63), Al 11.51–1804.33 µg/L, Cr ND–44.02 µg/L; seasonal totals up to 326.09 mg/L at one station.	Toxicity, bioaccumulation; Cr/Ni low carcinogenic risk at some sites.	[45]
Plastics (macro to micro, total load)	Mixed polymers (PE, PP, PS, PET, PVC, etc.)	Mass balance in a WWTP: plastics of all sizes tracked; macroplastics contributed more to total plastic mass than microplastics in the plant; total plastic mass flux quantified across screenings, sludge, and effluent.	Macro items dominate mass; operational implications for screening/sludge handling.	[46]
Synthetic dyes (textile effluents)	Reactive Black 5 (RB5), Reactive Blue 19; Direct Blue 71 (examples)	Real textile effluent (Tanzania): RB5 measured ~377.6 mg/L in raw wastewater before treatment. General textile wastewater characteristics: dye concentrations frequently 10–200 mg/L, depending on process/season (collated from plant measurements).	Color, toxicity; inhibits photosynthesis; treatment challenging.	[47,48]
Microplastics (MPs)	Fibers & fragments (PE, PP, PET, PS, PA, etc.)	Advanced WWTP (France): influent 109–1583 particles L^−1^ (≈61.5–100 µg L^−1^ by mass); removal 99.1–99.9%; effluent typically ~1–2 particles L^−1^ after tertiary steps. Year-long effluent analysis (Germany, 2 WWTPs): persistent MP detection in effluents (FTIR/Py-GC/MS, counts per L reported across seasons).	MPs pass conventional treatment in small numbers; sludge is a major sink.	[49,50]
Pathogens (microbes & viral RNA)	Enteric viruses (e.g., SARS-CoV-2 RNA), bacteria (e.g., *E. coli*, *Enterococcus*), protozoa	Municipal wastewater time-series: SARS-CoV-2 RNA consistently detected; wastewater concentration trends mirrored clinical case dynamics in the community (New Haven, USA).	Public health surveillance: infection risk in untreated flows & CSOs.	[51]
Pharmaceuticals & personal-care products (PPCPs)	Antibiotics (ciprofloxacin, sulfamethoxazole), analgesics (ibuprofen, diclofenac), beta-blockers, anticonvulsants	Municipal WWTPs (global review of original studies): typical ng/L–µg/L in influents; partial removal; residues in effluents.	Ecotoxicity, antibiotic resistance selection.	[52]
PFAS (per- & polyfluoroalkyl substances)	PFOA, PFOS, PFHxA, PFBS, 6:2 FTS (examples)	Meta-analysis of WWTPs: effluent medians generally range from tens to hundreds of ng/L (compound-specific); WWTPs act as sources for many PFAS to surface waters.	Very persistent; bioaccumulation & toxicity concerns; hard to remove.	[53]
Nutrients	Ammonium (NH_4_^+^-N), Total Nitrogen (TN), Total Phosphorus (TP), phosphate	Typical municipal influent (example dataset): COD~420 mg/L, cBOD_5_~200 mg/L, NH_4_-N~24 mg N/L, PO_4_-P~2.5 mg P/L (pre-treatment).	Eutrophication and oxygen depletion: core drivers of algal blooms.	[54]
Petroleum hydrocarbons/surfactants	LAS (linear alkylbenzene sulfonates), nonylphenol ethoxylates; TPH	(Values vary widely by catchment/industry; mg/L levels for surfactants in strong domestic/industrial influent are reported across plants; effluents are typically lower after biological treatment.)	Toxic to aquatic life; endocrine activity (nonylphenols).	[55]
Nitrilotriacetic acid (NTA)	NTA (chelating agent in detergents)	Germany, full-scale WWTP influents: NTA detected at 40–400 µg/L, effluents 10–80 µg/L; removal efficiency 70–90%.	Strong chelator; mobilizes heavy metals in the environment.	[56]
Bisphenol A (BPA)	BPA (plasticizer, epoxy resins)	WWTPs in Spain: influent 0.06–7.1 µg/L, effluent 0.01–0.5 µg/L, ~90% removal.	Endocrine disruptor; persistent in sludge.	[57]
Triclosan (TCS)	Triclosan (antimicrobial in soaps, toothpaste)	U.S. municipal WWTPs: influent 1.8–5.3 µg/L, effluent 0.1–0.5 µg/L (90–95% removal; sorption to sludge major sink).	Antimicrobial resistance driver; toxic to algae.	[58]
Phenols/alkylphenols	Phenol, 4-nonylphenol (4-NP), octylphenol	Europe WWTP survey: nonylphenol in influents 0.2–10 µg/L, effluents 0.1–3 µg/L; also, phenol itself detected at µg/L levels.	Estrogenic activity; aquatic toxicity.	[59]
Pesticides	Atrazine, diazinon, chlorpyrifos, imidacloprid, glyphosate (examples)	U.S. WWTP influents (EPA survey): pesticides generally <1 µg/L, often ng/L range; diazinon up to 0.3 µg/L; atrazine 20–200 ng/L; imidacloprid 30–150 ng/L.	Chronic aquatic toxicity; endocrine disruption.	[60]

**Table 3:** Comparative synthesis of major wastewater treatment technology groups, highlighting their principal treatment role, typical performance, technology maturity, relative implementation cost, key operational constraints, and most appropriate application context.

Technology Group	Main Role/Target Pollutants	Typical Strength	Maturity Level	Relative Cost	Main Limitation	Best Use Context
Conventional biological treatment	BOD, COD, TSS, routine nutrient removal	Strong for bulk pollutants; weak for PFAS, many pharmaceuticals, and ARGs	High	Low–Medium	Limited for persistent micropollutants	Core municipal treatment
Tertiary polishing	Residual solids, pathogens, and partial micropollutant control	Improves discharge and reuse quality	High	Medium	Carbon regeneration, by-products, and chemical demand	Post-secondary polishing
Membrane systems (MBR/NF/RO)	Fine particles, pathogens, salts, PFAS, reuse-quality water	Very high separation efficiency	Medium–High	High	Fouling, brine/concentrate disposal, energy demand	High-quality reuse and PFAS control
Advanced oxidation processes	Recalcitrant organics, pharmaceuticals, dyes, EDCs	High degradation potential	Medium	High	Energy use, oxidant demand, and possible toxic by-products	Targeted micropollutant polishing
Nature-based systems	Nutrients, solids, pathogens, some organics	Good low-energy removal with ecological co-benefits	Medium–High	Low	Land requirement, climate/season sensitivity, variable trace-contaminant control	Decentralized and low-resource settings
Bioenergy/resource-recovery systems	COD removal with methane/nutrient recovery	Strong circular-economy potential	Medium–High	Medium–High	Process control and polishing are often still needed	Plants seeking energy or nutrient recovery
Nano-enabled systems	Dyes, metals, antibiotics, and difficult micropollutants	Often excellent lab/pilot removal	Low	Medium–High	Scale-up uncertainty, leaching risk, lifecycle concerns	Specialized polishing, mainly pilot/research
Hybrid multi-barrier systems	Broad-spectrum pollutant control	Most robust overall performance	Medium	Medium–High	Greater design and operating complexity	Best where high effluent quality is required

**Table 4:** Representative conventional, advanced, biological, and hybrid wastewater treatment technologies, showing treatment principles, wastewater type or scale, characteristic performance, major advantages, and main practical limitations.

Method	Green Principle	Wastewater Type/Scale	Key Findings (Typical)	Citations
Upflow Anaerobic Sludge Blanket (UASB)	High-rate anaerobic digestion with granular sludge → methane (biogas) production	High-strength industrial & municipal; full-scale, widely deployed	Compact footprint; high COD removal for soluble wastes; strong methane recovery; granule formation critical for performance	[90]
Anaerobic Membrane Bioreactor (AnMBR)	Anaerobic digestion + membrane separation (retains biomass)—methane + high quality effluent	Municipal/industrial; pilot → demonstration	Very high COD removal, methane recovery; great for energy recovery and reuse-quality water; membrane fouling and temperature are key constraints	[91]
Aerobic granular sludge (AGS; Nereda^®^ technology, Royal HaskoningDHV, Amersfoort, the Netherlands)	Dense, fast-settling aerobic granules that combine simultaneous C/N/P removal in compact reactors	Municipal & industrial; full-scale Nereda installations exist	Compact footprint, reduced aeration/energy per load vs. conventional AS, simultaneous nutrient removal; now mature at full scale (Nereda^®^)	[92]
Mainstream Partial Nitritation–Anammox (PN/A)	Shortcut nitrogen removal—convert NH_4_^+^ → NO_2_^−^ (partial nitritation) then Anammox to N_2_ (autotrophic)	Municipal mainstream (low-strength)—pilot & demonstration	Very low external C demand and major aeration savings if stable; mainstream (low temp, variable load) remains challenging, but pilots show promise	[93]
Microbial Fuel Cells (MFC)/bioelectric systems	Microbes oxidize organics and generate electrons—electricity + treatment	Domestic/industrial; lab → small pilots	Demonstrated COD removal with low-power electricity generation; promising for decentralized low-energy systems; scale & power density remain limiting	[84]
Hybrid AnMBR + Constructed Wetland (CW)/AnMBR polishing	High-rate anaerobic energy recovery + nature-based polishing (CW) for nutrients & solids	Municipal/industrial; pilot → field demonstrations	AnMBR provides COD reduction + methane; constructed wetland polishes residual nutrients, solids, and micropollutants—attractive for decentralized/low-cost reuse chains	[94]
Mainstream anaerobic + partial aerobic staged treatment (Anoxic/Anammox hybrids)	Process intensification by staging (anaerobic for energy + aerobic/partial aeration for polishing)	Municipal; pilot/demonstration	Integrating anaerobic pre-treatment (AnMBR/UASB) reduces loading to the aerobic stage, increases net energy recovery; often followed by PN/A polishing or a constructed wetland	[95]
Anaerobic co-digestion/high-rate digesters + resource capture	Co-treat organic waste/wastewater to increase methane yield & stabilize organics	Industrial/agro + municipal co-digestion; full/pilot	Increased biogas yield and improved carbon balance; attention to inhibition (LCFAs, ammonia) and mixing/hydrodynamics needed	[96]
Bioelectro-anMBR/MFC-AnMBR hybrids (bioelectric intensification)	Couple bioelectrochemical cells with anaerobic reactors to enhance degradation/accelerate start-up	Agricultural/domestic pilots; lab → pilot	Reported faster start-up, improved COD removal, and combined energy recovery in pilot tests; integration technical complexity is increasing	[97]

**Table 5:** Innovative bioenergy and resource recovery approaches in wastewater treatment, highlighting technology characteristics, performance indicators, sustainability benefits, and circular economy relevance.

Method/Category	Main Advantages	Main Disadvantages/Challenges	Typical Performance Parameters	Best-Suited Wastewater Type/Scale	Key Sustainability Aspects	Why/When to Choose Next Step
Constructed Wetlands (CW) (H/V/Hybrid)	Low-cost, nature-based, robust; good nutrient & pathogen removal; landscape integration	Requires a large land area; performance fluctuates with season/climate; limited micropollutant removal	BOD/COD: 70–90%; TSS: 80–90%; TN: 40–70%; TP: 30–60%	Municipal, domestic, peri-urban communities	Low O&M, ecosystem co-benefits	Choose when land is available, long-term, low-cost, and ecological co-benefits are desired
Floating Treatment Wetlands (FTW)	Easy retrofit in ponds/lakes; high N/P uptake; adds aesthetics	Root clogging; limited for high-strength wastewater; requires harvest	TN removal: 40–70%; TP: 30–60%	Stormwater, small ponds, polishing	Ecosystem integration	Choose for polishing stormwater or ponds where conventional plants are impractical
Duckweed/Lemna Systems	Fast growth, biomass valorization (feed, bioethanol); high nutrient uptake	Sensitive to temperature, predators, toxins; monoculture risk	TN: up to 70–90%; TP: 60–80%	Agricultural runoff, digestate, and small-scale municipal	Circular economy (feed/biofuel)	Choose when nutrient valorization and circular economy are priorities
High-Rate Algal Ponds (HRAP)	Low-cost, O_2_ self-generation, biomass for biofuels; high N & P removal	Requires high sunlight and temperature control; algal harvesting is costly	COD/BOD: 60–80%; TN: 50–70%; TP: 40–60%	Domestic/municipal; warm climates	Biofuel co-production	Choose when solar resource is high & algal biomass recovery is possible
Vermifiltration	Earthworms + microbes; high BOD/COD and pathogen removal; simple O&M	Sensitive to toxins, temperature extremes	BOD/COD: 70–90%; Pathogen ↓ 2–3 log	Domestic/agro-industrial	Low-cost, decentralised	Choose small-scale decentralized wastewater, especially in rural areas
Bioflocculants	Biodegradable, replaces chemical alum/PAC	Large-scale production is not yet cost-effective	Turbidity/SS removal > 80%	Industrial & municipal	Avoids chemical sludge	Choose when the eco-friendly coagulant demand is high
Immobilized Enzymes (e.g., laccase)	High specificity for dyes/phenols; reusability	Enzyme cost, stability, and immobilization challenges	Color removal: >90%; phenols ↓	Textile, pharma	Green catalytic degradation	Choose for industrial effluents rich in recalcitrant organics
UASB (Anaerobic Digestion)	Energy recovery (biogas); compact footprint; low sludge	Lower nutrient removal; requires polishing	COD removal: 70–90%; CH_4_ yield: 0.2–0.3 m^3^/kg COD	High-strength industrial, municipal (warm climates)	Energy-positive, low sludge	Choose when high-strength wastewater + biogas recovery is desired
AnMBR	Produces clean water + methane; compact; high COD removal	Membrane fouling, cost, requires skilled O&M	COD removal > 95%; methane recovery high	Municipal/industrial	Energy recovery + water reuse	Choose when high effluent quality & reuse is a priority
Aerobic Granular Sludge (AGS)	Compact, simultaneous N/P removal; energy-efficient vs. CAS	Startup time requires controlled operation	COD/BOD: 80–90%; TN: >70%; TP: >70%	Municipal full-scale	Energy-saving, smaller footprint	Choose modern urban WWTPs with land/energy constraints
PN/A (Partial Nitritation-Anammox)	Low O_2_ & no external C; huge energy saving for N removal	Requires stable conditions; sensitivity to temp/DO	N removal efficiency 70–90%	Municipal & industrial	Low-carbon N removal	Choose for N-rich, C-poor wastewater with stable operation
MFCs (Microbial Fuel Cells)	Simultaneous COD removal + electricity	Low power density; scaling issues	COD: 60–80%; Power: 0.1–2 W/m^2^	Lab/pilot only	Energy recovery + sensing	Choose for niche: remote sensing + polishing
Hybrid AnMBR + CW	Combines high COD removal + polishing; energy balance	Larger footprint; more complex design	COD removal > 95% + nutrients ↓	Municipal/industrial	Integrative sustainability	Choose when energy recovery + natural polishing are both needed
Green Nanoparticles (Ag, ZnO, TiO_2_)	High reactivity; effective at low doses; solar-driven	Nanotoxicity concerns: cost of scaling	Dye/phenol removal > 90%; antibacterial action	Textile, pharma, lab → pilot	Green chemistry, photocatalysis	Choose for industrial recalcitrant effluents, if safe disposal is ensured
Magnetic Biochar/Graphene/Carbon Dots	Renewable feedstock; recyclable adsorbents/catalysts	Stability/recovery costs; possible leaching	Adsorption q_max: 100–500 mg/g; photocatalysis visible-light active	Industrial, dye/metal wastewater	Waste-to-resource	Choose when adsorbent regeneration and the circular economy are desired
Photocatalytic Membranes/Solar AOPs	Combine filtration & degradation; sunlight use	High capital cost; requires clear water for AOPs	COD/Pharma removal: 80–95%	Municipal/industrial polishing	Renewable-driven	Choose for micropollutant removal in advanced treatment
Renewable-Powered EC (solar-EC)	Uses PV or solar-thermal; reduced CO_2_	Dependence on solar; electrode fouling	Turbidity/metal removal > 90%	Textile/industrial	Energy + treatment synergy	Choose when solar potential is high + industrial wastewater with metals/dyes

**↓** The down arrow shows a reduction in its common practice in science.

**Table 6:** Green-synthesized nanoparticles and nanocomposite-based systems for wastewater treatment, summarizing biological sources, target pollutants, treatment matrices, and reported applications.

Title	Nanoparticle	Green Source	Target Pollutant(s)	Matrix	Citations
Green-synthesized AgNP-enhanced nanofiltration membranes for oily wastewater	Ag	*Hibiscus* extract	Oil, salts, dyes	Oily wastewater	[99]
Green synthesis of AgNPs using *Acacia ehrenbergiana* for RhB removal	Ag	Acacia cortex	Rhodamine B	Dye solution	[100]
Green ZnO NPs using *Phoenix dactylifera* waste for dye degradation	ZnO	Date waste	Textile dyes, bacteria	Wastewater	[101]
Green ZnO from algae (*Padina pavonica*) for MB removal	ZnO	Algae	Methylene blue	Dye solution	[102]
Green synthesis of TiO_2_ using *Impatiens rothii* extract	TiO_2_	Impatiens leaves	Dyes (RhB)	Dye solution	[103]
TiO_2_ NPs from *Syzygium cumini* for Pb removal	TiO_2_	*Syzygium cumini*	Pb (II)	Industrial wastewater	[104]
*Bacillus subtilis*-mediated TiO_2_ NPs for dye removal	TiO_2_	*Bacillus subtilis*	Dyes	Dye solution	[105]
Green TiO_2_ via *Acorus calamus* for RhB degradation	TiO_2_	*Acorus calamus*	Rhodamine B	Dye solution	[106]
CuO NPs for tanning wastewater sterilization & dye removal	CuO	Plant extract	Microbes, dyes	Tanning wastewater	[107]
Aloe vera-derived CuO NPs for contaminants	CuO	*Aloe vera*	Dyes/contaminants	Model solutions	[108]
CuO via green combustion for dye degradation	CuO	Plant extract	Dyes	Dye solution	[109]
Plant-based CuO NPs for tanning wastewater	CuO	Plant extract	Metals, microbes	Tanning wastewater	[110]
CuO from *Seriphidium oliverianum* for dyes	CuO	Seriphidium	Dyes	Dye solution	[111]
CuO via *Parthenium hysterophorus* for rifampicin	CuO	Parthenium	Rifampicin (antibiotic)	Antibiotic solution	[112]
nZVI from eucalyptus leaves for eutrophic wastewater	nZVI (Fe^0^)	Eucalyptus	Nutrients, organics	Wastewater	[113]
Montmorillonite-supported nZVI via green tea for Cr (VI)	nZVI	Green tea	Cr (VI)	Water & soil	[114]
Tea-leaf-derived nZVI for dye Fenton degradation	nZVI	Tea leaves	Mixed dyes	Dye solution	[115]
*Ricinus communis*-based nZVI for MB removal	nZVI	*Ricinus communis*	Methylene blue	Dye solution	[116]
Fe_3_O_4_ via tea polyphenols for dye removal	Fe_3_O_4_	Green tea	Dyes	Dye solution	[117]
Fe oxide NPs via *Psidium guajava* leaves	Fe_2_O_3_/Fe_3_O_4_	*Psidium guajava*	Dyes, pathogens	Dye solution	[118]
Magnetic Fe_3_O_4_ via *Cordia myxa* extract	Fe_3_O_4_	*Cordia myxa*	Dyes, metals	Model solution	[119]
*Spirulina*-based iron oxide NPs for dyes	Fe oxide	*Spirulina platensis*	Crystal violet, methyl orange	Real effluent	[120]
*Clove*/coffee-extract Fe oxide NPs for Cd/Ni	Fe oxide	*Clove* & coffee	Cd (II), Ni (II)	Aqueous solutions	[121]
Biosynthesized AgNPs using *Cestrum nocturnum* for dye degradation	Ag	*Cestrum nocturnum*	MB, Congo Red, 4-NP, 4-NA	Dye solution	[122]
Green ZnO NPs via *Sargassum muticum* (marine brown algae)	ZnO	*Sargassum muticum*	MB, bacteria	Aqueous solution	[123]
Review: Efficient dye degradation using green ZnO-based nanoplatforms	ZnO (review)	Various	Dyes	-	[124]
Green synthesis of ZnO using *Justicia adhatoda* for MG & 4-NP degradation	ZnO	*Justicia adhatoda*	Malachite Green, 4-NP	Dye solution	[125]
Eco-friendly AgNPs + TiO_2_/ZnO mix for textile dye degradation	Ag + TiO_2_/ZnO	Chitosan biopolymer	Acid Red 37	Dye solution	[126]
Ag–Mn oxide NPs for malachite green degradation	Ag–Mn oxide	Wet chemical	Malachite Green	Dye solution	[127]
Review: Catalytic dye removal by green-synthesized SeNPs	SeNPs (review)	Various	Dyes	-	[128]
Biogenic ZnO-NPs for municipal wastewater & wheat cultivation	ZnO	*Shewanella* sp.	Nutrients, COD, phosphate	Municipal wastewater	[129]
Biogenic AgNPs for textile wastewater	AgNPs	Conocarpus + *Pseudomonas*	Reactive Black 5, Reactive Red 120	Textile wastewater	[130]
Magnetite-pectin nanoparticles for levofloxacin removal	Fe_3_O_4_	Citrus pectin	Levofloxacin	Wastewater	[131]
Natural product-coated magnetite NPs for mixed wastewater	Fe_3_O_4_	*Jatropha curcas*, *C. tamala*	Dyes, metals, bacteria	Mixed wastewater	[132]
FeNPs from bioflocculant for coal-mine wastewater	Fe NPs	Bioflocculant (Actinomycete)	BOD, COD	Coal mine wastewater	[133]
Filter paper fabricated with green-tea Fe NPs	Fe NPs	Green tea	Heavy metals	Industrial wastewater	[134]
Nano-bioremediation of textile wastewater using myco-CuO-NPs	CuO	*Fusarium oxysporum*	Dyes, Pb, Cr, Ni	Textile wastewater	[135]
Green CuO NPs for Pb, Ni, Cd removal	CuO	Mint & orange peel	Pb, Ni, Cd	Industrial water	[136]
Domestic wastewater disinfection filter—biosynthesised AgNPs	AgNPs	*Streptomyces* sp.	Microbes (coliforms)	Domestic wastewater	[137]
Chitosan–magnetite composite for fluoroquinolone removal	Fe_3_O_4_ + chitosan	Chitosan	Ciprofloxacin, levofloxacin	Aquaculture wastewater	[138]

**Table 7:** Green-synthesized nanocomposites and photocatalytic membrane technologies for multifunctional wastewater treatment, including composition, synthesis strategy, pollutant-specific applications, and practical relevance.

Title	Nanocomposite/Composition	Application/Pollutant	Citations
Green-synthesized rGO/TiO_2_ nanocomposite using carob extract for heavy metal and dye removal	rGO + TiO_2_ (green, carob extract)	Adsorption of Zn^2+^, Ni^2+^, Pb^2+^, Cd^2+^; photocatalytic degradation of methylene blue	[139]
CuO@A-TiO_2_/Ro-TiO_2_ nanocomposite (green synthesis) for photocatalytic degradation of dye in textile wastewater	CuO + Anatase-TiO_2_ + Rod-TiO_2_	Photocatalytic elimination of Reactive Orange 16 in actual textile wastewater	[140]
Nanocomposites from spent coffee grounds with FeO/ZnO nanoparticles (green)	FeO + ZnO supported on spent coffee grounds	Treatment of textile wastewater (dye removal, etc.)	[141]
Green synthesis of CuO/PANI nanocomposite for Pb(II) removal from contaminated water	CuO nanoparticles in Polyaniline (PANI)	Adsorption of Pb^2+^ ions from water	[142]
MMT/Ag (montmorillonite–silver) nanocomposite synthesized via weed extract for dye removal	Montmorillonite clay + Ag NPs	Adsorption of methylene blue dye from aqueous solution	[143]
Novel wastewater treatment by using newly prepared green seaweed–zeolite nanocomposite	Seaweed (bio-waste) + Zeolite	Removal of organic/inorganic contaminants in wastewater	[144]
Eco-friendly, sustainable, synthesised nanocomposite (corn leaves + ZnO) for heavy metal removal	Corn leaves + ZnO	Removal of Fe^2+^ and Ni^2+^ from water	[145]
Metal-based green-valorised nanocomposites for dye remediation (e.g., ZnO/biochar, nano-zerovalent manganese/biochar)	ZnO/biochar, Mn^0^/biochar	Adsorptive removal of dyes (e.g., Congo red) from water	[146]
Synthesis of hybrid nanostructures by green methods (e.g., Cu@TiO_2_ leaf extract composites) for wastewater remediation	Cu@TiO_2_ via Cedrus deodara; Ni@Fe_3_O_4_ and CuO NPs via Euphorbia extract	Photocatalytic degradation of organic pollutants in wastewater	[147]
Review of polymeric nanocomposites for photocatalytic wastewater treatment	Various polymer-based nanocomposites	Photocatalytic degradation of dyes and organic pollutants	[148]
ZnO/rGO nanocomposites synthesized using red rice husk extract for dye photodegradation	ZnO + reduced graphene oxide (rGO) via red rice husk extract	Photocatalytic degradation of malachite green	[149]
Pd/Fe_3_O_4_ nanocomposite via Hibiscus extract for reductive catalysis of Cr(VI) and 4-nitrophenol	Pd on Fe_3_O_4_, green Hibiscus extract synthesis	Catalytic reduction of Cr(VI) and 4-NP in water	[150]
Chitosan-based nanocomposite gel with TiO_2_ and silica for dye removal	Chitosan + silica + TiO_2_ NPs	Adsorption of methylene blue; Cr(VI) adsorption potential	[151]
ZnO/NiFe_2_O_4_ nanocomposite synthesized via green route for dye degradation	ZnO (green) + NiFe_2_O_4_	Photocatalytic degradation of methylene blue in wastewater	[152]
MgO/graphene nanoplatelet nanocomposite for photocatalytic wastewater purification	MgO + graphene nanoplatelets	Photocatalytic degradation of pollutants in industrial wastewater	[153]

**Table 8:** Green Nanotechnology, Nano-Bio Hybrids, and Renewable-Energy-Driven Advanced Processes for Sustainable Wastewater Treatment: Principles, Applications, and Representative Studies.

Method	Green Principle	Wastewater Type/Scale	Key Findings	Citations
Green-synthesized Ag nanoparticles	Plant/biomass extracts act as reducing/stabilizing agents; antimicrobial + catalytic dye reduction	Textile dye, hospital effluent; lab → pilot	Rapid dye reduction, strong antibacterial action, and easy synthesis from waste plant extracts	[100]
Green ZnO nanoparticles	Phytosynthesis of ZnO NPs; visible/UV photocatalysis	Dye wastewater; lab	High photocatalytic degradation under sunlight/UV; bandgap tuning via extracts	[154]
Green TiO_2_/MnO_2_ heterojunctions (biogenic)	Plant-extract reduction/templating → heterojunction photocatalyst; solar-driven	Industrial (pulp & paper) effluent; pilot membrane tests	Strong sunlight-driven photocatalysis + membrane separation; large COD reduction in trials	[155]
Green ZnO–TiO_2_/other mixed oxide composites	Biogenic doping/heterojunctions → reduced recombination, visible response	Dyes, pharmaceuticals; lab/pilot	Faster pollutant mineralization under solar irradiation vs. single oxide	[156]
Magnetic biochar nanocomposites (Fe_3_O_4_@biochar)	Biomass-derived adsorbent + Fe_3_O_4_ for magnetic recovery	Dye/metal wastewater; batch/column pilot	High adsorption capacity; easy magnetic separation & reusability	[157]
Nanocellulose membranes/adsorbents	Renewable cellulose → high-surface, mechanically robust membranes/adsorbents	Dye/heavy metal; lab → pilot	Exceptional mechanical strength, tunable surface chemistry, and high flux/selectivity	[158]
Biomass-derived carbon dots/carbon quantum dots (CDs/CQDs)	Hydrothermal transformation of agrowaste → photoluminescent photocatalysts & sensors	Dye & micropollutant lab tests	Visible-light photocatalysis, low-toxicity sensors, and facile synthesis from waste	[159]
Photocatalytic membranes (green NP-embedded)/Photocatalytic membrane reactors (PMR)	Embed green NP photocatalysts in membranes → filtration + degradation	Tertiary polishing; pilot	Simultaneous particle removal and decomposition of organics; reduced fouling via photocatalysis	[160]
Solar-driven AOPs (solar photo-Fenton, solar/UV-H_2_O_2_, solar/persulfate)	Use sunlight to activate oxidants/photocatalysts → minimal grid energy/chemicals	Municipal effluent/industrial recalcitrant wastewater; pilot	High removal of micropollutants & pathogens in pilot trials; pH/iron management matters	[161]
Renewable-powered electrocoagulation (PV-EC/solar-EC)	Photovoltaic electricity drives EC cells—coagulation without grid fossil electricity	Textile/dye, municipal; pilot	Effective turbidity/dye/metal removal; favourable LCA if PV sized appropriately; battery-less direct-PV schemes possible	[162]
Green-synthesized CuO/Cu_2_O nanoparticles & nanocomposites	Plant-assisted Cu-oxide NPs: low cost, strong photocatalysis & Fenton-like activity	Dye/antibiotic wastewater; lab	Good visible-light activity and bactericidal properties; Cu-leaching management required	[163]
Bio-MOFs/biomass-templated porous materials	Biomass templates → metal–organic frameworks/activated MOF-like structures for adsorption & catalysis	Pharmaceuticals, dyes; lab/pilot	Extremely high surface area & tunable functionality; selective adsorption of pharmaceuticals/metal ions	[164]
Graphene oxide/reduced GO from biomass & composites	Convert agrowaste → GO/rGO for adsorption, catalytic composite supports	Dye/metal wastewater; lab/column	High adsorption & excellent electron transfer when combined with photocatalysts; regeneration possible	[165]
Photocatalytic aerogels & 3D porous photocatalysts	Low-density 3D structures with immobilized green NPs → high contact area	Industrial effluents; lab/pilot	High throughput, easier recovery than slurry photocatalysts; good sunlight utilization	[166]
Magnetic photocatalysts (Fe-doped TiO_2_, Fe_3_O_4_-TiO_2_)	Magnetic recovery + photocatalysis (green synthesis pathways exist)	Dye/aqueous organics; lab → pilot	Combine high photocatalytic rates with straightforward magnetic separation	[167]

**Table 9:** (**A**) Green Nanomaterials for Water and Wastewater Treatment: Sources, Applications, One Health Relevance, and Links to Cancer Prevention and Therapy. (**B**) Comprehensive Toxicological Analysis of Lead (Pb) and Chromium (Cr): Carcinogenic Pathways, Affected Genes/Proteins, and Associated Cancer Types in Humans.

(**A**)							
**Green Nanomaterial**	**Source/Synthesis**		**Water/Wastewater Application**		**One Health Relevance**	**Cancer Link**	
Chitosan nanoparticles	Derived from crustacean shells, green synthesis		Adsorption of heavy metals (As, Pb, Cd)		Reduces toxic metals in water, protecting humans, animals, and ecosystems	Removes arsenic and lead, linked to skin, bladder, and lung cancers	
Silver nanoparticles (AgNPs)	Plant extract-mediated synthesis		Antimicrobial treatment of wastewater		Reduces pathogenic bacteria and AMR spread	Potential for anticancer therapy via selective cytotoxicity	
Zinc oxide nanoparticles (ZnO NPs)	Plant-based or microbial synthesis		Photocatalytic degradation of organic pollutants (dyes, pesticides)		Minimises chemical pollution in ecosystems	Degrades carcinogenic dyes, reducing exposure risk	
Gold nanoparticles (AuNPs)	Green synthesis using plant extracts		Targeted removal of pollutants or drug delivery		Non-toxic, safe for environmental and biomedical use	Can deliver anticancer drugs or induce apoptosis in tumour cells	
Graphene oxide/Carbon dots	Biomass or food waste		Adsorption of dyes, heavy metals, and pharmaceutical residues		Reduces persistent pollutants, protecting environmental and animal health	Some carbon-based nanomaterials exhibit anticancer activity in vitro	
Titanium dioxide nanoparticles (TiO_2_ NPs)	Plant-mediated green synthesis		Photocatalytic degradation of organic pollutants and disinfectants		Reduces chemical and microbial contamination in water	Removes carcinogenic dyes and toxins from water	
Copper nanoparticles (CuNPs)	Microbial or plant extract synthesis		Antimicrobial treatment of wastewater		Helps control pathogens, reducing risk to humans and animals	Potential cytotoxic effects on cancer cells in research studies	
(**B**)							
**Metal**		**Cancer Types**		**Affected Genes/Proteins**			**Reference**
Lead (Pb)		Lung cancer		IL-1β, TNF-α, IL-6; oxidative-stress markers; pathways linked to DNA damage/repair disruption			[168]
Lead (Pb)		Breast cancer		BCL2, p53; markers of genomic instability (MSI, TMB); epigenetic alterations (DNA methylation, histone changes)			[169]
Lead (Pb)		Kidney (renal) cancer		δ-ALA dehydratase (δ-ALAD), ferrochelatase; oxidative-stress and heme-biosynthesis disruption			[170]
Chromium (Cr, hexavalent Cr(VI))		Lung cancer (incidence & mortality)		p53, MLH1, MSH2, BCL2, VEGF; activation of NF-κB pathways; DNA-protein crosslinks and oxidative DNA damage			[171]
Chromium (Cr, hexavalent Cr(VI))		Lung—mechanistic (preclinical + human bioinformatics)		ABHD11-AS1 (lncRNA), USP15, TRAF3, RelB, PD-L1 (CD274), IL-6/JAK-STAT3; non-canonical NF-κB activation			[172]
Chromium (Cr)		Larynx, bladder, kidney, testicular, bone, thyroid cancers (associations reported)		ATM, ERK, c-MYC, XPF, Bcl-X; genes involved in DNA repair, apoptosis, and cell-cycle regulation			[169]
Chromium (Cr)		Gastric & colorectal cancers (limited/weak evidence)		Genes/pathways related to oxidative stress and DNA-repair disruption (epigenetic changes also reported)			[169]

## Data Availability

No new data were created or analysed in this study. Data sharing is not applicable to this article.
